# Early Nutrition and Risk of Type 1 Diabetes: The Role of Gut Microbiota

**DOI:** 10.3389/fnut.2020.612377

**Published:** 2020-12-23

**Authors:** Elvira Verduci, Chiara Mameli, Matilde Amatruda, Agnese Petitti, Sara Vizzuso, Farah El Assadi, Gianvincenzo Zuccotti, Shaikha Alabduljabbar, Annalisa Terranegra

**Affiliations:** ^1^Department of Pediatrics, Vittore Buzzi Children's Hospital, University of Milan, Milan, Italy; ^2^Department of Health Sciences, University of Milan, Milan, Italy; ^3^College of Health and Life Sciences, Hamad Bin Khalifa University, Doha, Qatar; ^4^Research Department, Sidra Medicine, Doha, Qatar

**Keywords:** T1D, early nutrition, gut microbiota, probiotics, prebiotics, post-biotics

## Abstract

Type 1 diabetes (T1D) appears most frequently in childhood, with an alarming increasing incidence in the last decades. Although the genetic predisposition is a major risk factor, it cannot solely explain the complex etiology of T1D which is still not fully understood. In this paper, we reviewed the most recent findings on the role of early nutrition and the involvement of the gut microbiota in the etiopathogenesis of T1D. The main conclusions that are withdrawn from the current literature regarding alleviating the risk of developing T1D through nutrition are the encouragement of long-term breast-feeding for at least the first 6 months of life and the avoidance of early complementary foods and gluten introduction (before 4 months of age) as well as cow milk introduction before 12 months of life. These detrimental feeding habits create a gut microbiota dysbiotic state that can contribute to the onset of T1D in infancy. Finally, we discussed the possibility to introduce probiotics, prebiotics and post-biotics in the prevention of T1D.

## Introduction

Diabetes is a serious issue tackled globally. It's considered one of the top 10 causes of death in adults. According to the International Diabetes Federation Atlas, in 2019, the number of people with diabetes was ~463 million (1). Children represent 5–15% of total diabetic patients (2). Type 1 diabetes (T1D) is an autoimmune disease resulting from the destruction of insulin-producing β-cells in the pancreas which is promoted by T-cells (3), producing autoantibodies. Although the disease can occur at any age, T1D develops mostly in youth as 85% of all cases worldwide are diagnosed in individuals under 20 years of age (4). Results from EURODIAB study held in 22 European countries showed that T1D incidence rate in children under 14 years old increased by 3.4% annually from 1989 to 2013 (5). A similar trend is reported by the Centers for Disease Control and Development (CDC) in the United States describing increasing incidence of T1D by 1.9% per year between 2002 and 2015 in children younger than 20 years old (6).

The etiology of T1D is complex and not fully understood; the genetic susceptibility along with environmental triggers can contribute to the development of the disease (7). Genetics play a crucial role in acquiring T1D as supported by familial inheritance studies; however, the inheritance pattern is complex and unclear (8). The risk of developing T1D is enhanced in individuals with multiple first-degree relatives affected by the disease (9). Besides, T1D is more common in males than in females (10), and children of fathers with T1D are more likely to get the disease than those who have mothers with T1D (11). However, among all children with genetic risk to develop diabetes, only a 5% of them develop the disease (12). The potential role of environmental factors is suggested by many facts: the increasing incidence of T1D in industrialized countries and in young children (13, 14); the low concordance among homozygotic twins (15); and the evidence that moving from a low incidence to a high incidence region increases the risk of the disease (16). Environmental factors that could trigger T1D progression in children are numerous: viral infections (8, 17, 18), obesity (19, 20), lack of exercise, puberty, rapid longitudinal growth, psychological stress (19), diet and particularly high glycemic index diets (21), vitamin D deficiency and low diversity of gut microbiome (8, 19).

Currently, there is no clear evidence of the impact of nutritional and environmental factors in the development of autoimmunity and T1D. Understanding their pathogenetic role could be a crucial strategy for the prevention of the disease in early infancy. In this review we will discuss the most recent findings on the role of early nutrition and the involvement of the gut microbiota in the etiopathogenesis of T1D.

## Early Nutrition and Its Role in T1D

Recent studies show that β-cells autoimmunity develops in the first years of life; indeed, in most cases autoantibodies can be detected by 2 years of age (14). This led researchers to look for environmental factors that act early in life, and special attention has been directed toward nutritional factors (22).

Many retrospective and prospective studies have been conducted to define the role of dietary factors in the development of T1D; however, results are still controversial. The inconsistency of these results might also reflect the influence of genetic background on the individual susceptibility to external factors. Moreover, most of these studies focused on adult diet, whereas recent researches highlighted the role of infant feeding practices and early nutrition in the development of T1D (23). Hereafter, we will review and discuss the available evidence about the role of breastfeeding and the introduction of complementary and single foods, such as cow milk and gluten, in the onset of T1D.

### Breastfeeding

Breastfeeding has several beneficial effects on maternal and child health (24), and many studies were performed to assess its potential impact on T1D ending in controversial results. Data from two large population-based cohorts of a total of 155,392 Danish and Norwegian children revealed that the risk of T1D doubled in those who were not breastfed. No significant difference was observed upon comparing the duration of breastfeeding (25). However, data from a meta-analysis of 43 studies, including 9,874 patients with T1D, showed that exclusive breastfeeding for >2 weeks is associated with a reduction in the risk of diabetes by 15% while a prolonged duration (>3 months) resulted in a weaker association. No association was found for non-exclusive breast-feeding independently from the duration (26). Similarly, conflicting results were obtained from previous prospective cohort studies assessing the link between breastfeeding and beta cells autoimmunity in children with genetic susceptibility to T1D (27).

There are many hypothetical mechanisms implicated in the protective effect of human milk. Breastfeeding has a central role in influencing gut microbiota and immunity. It contains nutrients and bioactive substances (cytokines, growth factors, immunomodulators, oligosaccharides) that promote the maturation of immune system and modulate its functions (16, 28, 29). A study conducted on diabetes-prone rats showed that breastfeeding decreases the number of activated lymphocytes and the production of pro-inflammatory cytokines (IL-4, IL-10, IFN-γ) while it increases the number of T regulatory cells (CD4+ CD25+ FoxP3+). In particular, long-term exclusively breastfed rats have reduced number of CD4+ T cells in the mesenteric lymph nodes and an expansion of both effector and natural T regs. Exclusive and prolonged breastfeeding reduced the risk of autoimmunity by limiting the introduction of external antigens and by shifting the balance between tolerogenic cells and autoreactive cells (30, 31). A recent research on non-obese diabetic mice (NOD-mice) showed that a 4-weeks supplementation with human milk oligosaccharides (HMOS) leads to a significant reduction in T1D incidence (29). Furthermore, human milk bacteriome (HBM) could play a role in immunoregulation through competitive exclusion of pathogenic bacteria and active production of antimicrobial and metabolic molecules (32). *In-vitro* studies demonstrated that HBM-derived *Lactobacilli* have anti-bacterial activity against Staphylococcus aureus *in vitro*, and it also inhibits adhesion of Salmonella enterica in infected mice (33). HBM inhibits anaerobic and facultative bacteria by producing acids and therefore lowering gut pH (34). HBM-derived *Lactobacilli* strains have immunomodulatory activity *in vitro* by modulating immune cell function, cytokines and chemokines. This effect was not observed with probiotic bacteria not derived from human milk (35). In addition, breast milk contains insulin that has a protective effect against autoimmunity as was proved in rats by driving the maturation of gut epithelium as well as in humans by downregulating production of IgG antibodies to bovine insulin (16). A case–control analysis of fatty acids serum concentration in infants with genetic susceptibility to T1D, within the Finnish Dietary Intervention Trial for the Prevention of Type 1 Diabetes (FINDIA), at the age of 3 and 6 months revealed that docosahexaenoic acid (DHA) levels were inversely associated to islet autoimmunity, while higher *n*-6:*n*-3 fatty acids ratio increased the risk. Moreover, the quantity of breast milk consumed per day was inversely associated with primary insulin autoimmunity, while the quantity of cow milk consumed per day was directly associated. Even if further studies are warranted to clarify the independent role of fatty acids in the development of T1D, omega-3 long chain polyunsaturated fatty acids consumed during breastfeeding might provide protection against type 1 diabetes-associated autoimmunity (36).

### Complementary Foods

According to World Health Organization (WHO) recommendations for infant feeding, exclusive breast-feeding represents the ideal nutritional strategy during the first 6 months of life for its beneficial effects on maternal and infant health (37). After 6 months of age, human milk alone is not sufficient to meet the energetic and nutritional needs of the baby. From this age on, the introduction of complementary foods is required to ensure the adequate infant growth and development. Any food can be offered by gradually increasing consistency and variety; however, sugars, salt and sugar-sweetened beverages should be avoided (38).

Evidence from systematic reviews revealed that complementary feeding initiation before 3–4 months of age is associated with higher risk of allergic conditions, while gluten introduction before 4 months can be linked to the development of celiac disease and type 1 diabetes mellitus (39). The Type 1 Diabetes Prediction and Prevention Project (DIPP), a prospective cohort study, aimed to evaluate the effect of complementary foods introduction on β cell autoimmunity. More than 3,000 newborn babies with genetic susceptibility to T1D were periodically screened for β cell autoimmunity seroconversion until 12 months of age. The study revealed that early introduction (between 3 and 4 months) of fruit, berries and roots was associated with a higher risk to develop β cells autoimmunity (27). Nevertheless, there is no consisting evidence that delaying the introduction of certain foods has a beneficial role in preventing T1D. Results are often discordant or inconclusive, and more data from large randomized controlled trials are needed.

### Cow Milk

According to current recommendations for infant feeding, cow milk introduction should be avoided before 12 months of life: early exposure has been linked to higher risk of developing allergy and to occult gastrointestinal blood (38). In the past years, different authors studied cow milk as potential trigger for T1D with discordant results, finding both an increased (40) or a decreased risk (41) of developing beta cells autoimmunity and T1D. Conversely, the TRIGR study on genetically susceptible children, found no protective effect of extensively hydrolyzed casein-based formula compared to a standard formula in the development of islet autoimmunity (42). To similar conclusions ended the prospective cohort study The Environmental Determinants of Diabetes in the Young (TEDDY study) which collected information on feeding pattern of 8,506 children with increased genetic risk for type 1 diabetes and founded no significant association with islet autoimmunity in infants fed with extensively hydrolysed compared to non-hydrolysed formula feeding (43). Cow milk proteins are known to have an intrinsic allergenicity particularly beta-lactoglobulin, bovine serum albumin, α-casein, κ-casein (44). Oral tolerance to cow milk antigens could be impaired in individuals with a genetic susceptibility to T1D, and this could trigger autoimmunity (45, 46). Bovine albumin and insulin have also been considered as possible triggers for autoimmunity given their similarity to endogenous pancreatic antigens (21, 30, 47). Immunological cross-reactivity between bovine proteins and beta cells' antigens could represent another hypothetical mechanism explaining the association between cow milk and T1D (48). Early exposure to cow's milk has been associated with increased gut permeability and altered barrier function, which predispose to exogenous antigen reactivity and immunologic dysregulation (49). Recent observations suggest that altered intestinal permeability could be a key step in the pathogenesis of the subclinical enteropathy underlying type 1 diabetes (50). It's worth to note the different position of fermented milk and dairy products known for the beneficial effect on human health (51). A recent systematic review on the effect of yogurt and fermented milk in infants and toddlers (0–24 months) confirmed the health benefits also in this age class, reporting positive effects on acute diarrhea as well as atopic dermatitis and food sensitivity. The same review reported the benefic effect on the gut microbiota that we discussed more in detail in Section Complementary Feeding-Induced Microbial Changes and Immune Response (52). No direct data have been found of the effect of the yogurt and fermented milk in T1D children.

### Gluten and Cereals

In the last decade, studies on rodents revealed a potential diabetogenic effect of gluten by inducing an immune dysregulation (53). According to the study of Funda et al., a gluten-free diet in NOD mice prevented the progression of T1D (54). Gluten proteins resist to enzymatic digestion and represent a constant immunologic trigger that can lead to immune dysregulation. This mechanism could explain gluten role in the similar pathogenic pathways of celiac disease and T1D (55). The Diabetes Autoimmunity Study in the Young (DAISY) examined dietary pattern of 1,835 infants at increased risk for diabetes (development of at least two specific autoantibodies in succession): islet autoimmunity and diabetes progression were not influenced by gluten cumulative amount in the first years of life, while introduction of gluten before 4 months was significantly associated with a higher risk of developing T1D (56). Nevertheless, previous prospective observational cohort studies could not find any link between the time of gluten introduction and islet autoimmunity (55). Accordingly, a small randomized controlled trial found no significant beneficial effect from delaying gluten introduction from 6 to 12 months (57).

The most recent opinion of experts regarding the appropriate age for introducing complementary foods concluded that gluten introduction before or after 6 months of age has neither beneficial nor negative effect on the risk of developing T1D. Gluten can be introduced between 4 and 12 months, but earlier introduction (before 4 months) is associated with the development of celiac disease in children at higher risk (56, 58). The prospective birth cohort FINDIA followed 6,081 infants with genetic susceptibility to type 1 diabetes up to 6 years and revealed that higher intake of oats, gluten-containing cereals and gluten (estimated trough 3 days food record) is associated with an increased risk of islet cell autoimmunity (59). The exposure to gluten was analyzed in a cohort od 6,605 children from the TEDDY study and the data showed that higher gluten intake during the first 5 years if life was associated with an increased risk for celiac disease (60). Further studies are needed to clarify the pathogenetic role of gluten and whether a gluten-free diet could be an essential component of medical nutrition therapy to prevent the onset and progression of T1D.

### Micronutrients

Increasing attention has been recently focused on the potential role of micronutrients intake during early life in etiology of T1D. As clearly outlined in a recent review, vitamin D and E and zinc are the most studied factors (22).

Vitamin D is commonly known for regulating of calcium and phosphate metabolism but also exerts several immunomodulatory effects on innate and adaptive immune system. Association between low levels of serum 25-hydroxyvitamin D have been linked to increased risk of many immune-related disorders, including T1D (61). Recently, Norris et al. described that higher serum levels of 25(OH) vitamin D are associated with lower risk of islet autoimmunity in children at increased genetic risk for T1D (62). However, the results of follow-up studies on children showed no significant association between vitamin D status at a pre-diagnostic stage and T1D progression later in life (63–65). Inconsistent results came from studies on maternal vitamin D supplementation for prevention of T1D in offspring (66). Moreover, a recent systematic review of randomized controlled trials suggests that vitamin D supplementation in both children and adults plays a role in the control of disease activity by reducing insulin requirement and stimulating C-peptide levels (67) but there is still no evidence of long-term effects of vitamin D early supplementation on T1D risk.

The action of vitamin A to control immune response has led to a growing hypothesis of potential role of vitamin A in T1D as an autoimmune disease. Vitamin A regulates the adaptive and innate immune responses by different mechanisms for example, by its ability to transform Th1 to Th2 lymphocytes. A recent review reported that both vitamin A and all-trans retinoic acid effectively induced immune tolerance that inhibited islet inflammation and progression to diabetes (68). Further studies are needed to evaluate the vitamin A modulating effect on the development of T1D.

Recent research has been focused on the potential role of oxidative stress induced by free radicals in the pathogenesis of T1D. Based on this assumption, micronutrients with antioxidant properties as vitamin C (ascorbic acid), zinc (Zn) and selenium (Se) may play a role in the pathogenesis and exacerbation of this disease. Mattila et al. examined plasma ascorbic acid concentration in children at high genetic risk of T1D (within the TEDDY study), initially at 6 and 12 months and then annually up to 6 years of age. The Authors found that higher plasma ascorbic acid levels were associated with decreased islet autoimmunity risk, but not with T1D risk progression (69).

Results from animal studies revealed that selenium-dependent proteins (seleno-proteins) with redox properties are involved in glucose metabolism, given that insulin release and signaling are influenced by the cellular redox potential (70). Significantly lower levels of Se and Zn were found in children affected by T1D and glycosylated hemoglobin (HbA1c) levels appeared inversely correlated with Se and Zn levels (71). Moreover, Zn concentration in drinking and stream water has been inversely associated to T1D (72, 73).

## The Potential Role of The Gut Microbiota As An Environmental Trigger for T1D

Consistent evidence from literature shows that the gut microbiota can influence the innate and adaptive immune system (74, 75), and many studies have highlighted the long-term impact of shaping the microbiota during early life on the immunity (76–80). The term “Microbiota” was first used in 2001 by Lederberg and McCray (81) and has been described as the “assemblage of microorganisms present in a defined environment” (82). The human body hosts up to 100 trillion symbiotic bacteria, primarily in the intestinal track, defined as the gut microbiota (83).

As for many other autoimmune diseases, the gut microbiota has been implicated in T1D pathogenesis through multiple mechanisms that involve the intestinal inflammation (49, 84), the epithelial barrier integrity (85, 86) and the modulation of tolerance to dietary antigens (87). The role of the gut microbiota in T1D pathogenesis was first discovered in animal studies on the Myd88^−/−^ NOD mice that developed T1D under germ-free conditions in contrast to pathogen-free conditions (88). Antibiotic treatment exhibited a prompting response for T1D development in the non-obese diabetic (NOD) mice by selectively eliminating specific bacterial species (89, 90). Interestingly, the antibiotic treatment had a more pronounced influence at the early age (from birth to day 28) compared to the mice that were given antibiotics starting 8 weeks of age until the disease's onset (91). Similarly, probiotics can alter the microbial profile by enhancing favorable bacterial species. In NOD mice (92–94) and bio-breeding diabetes-prone (BB-DP) rats (95), probiotics administration prevented T1D development. Dysbiosis, is hypothesized to be the underlying factor behind the triggered autoimmune response. A recent cohort study confirmed a microbial dysbiosis state in children who had multiple islet autoantibodies as well as children who were recently diagnosed with T1D; the reported dysbiosis was linked to permeability in the epithelial membrane (96). The microbiome in the duodenal mucosa showed a disease-specific manner in T1D patients, and it was associated with the expression of T1D-related genes (97). Consistently, taxonomic and functional differences in the gut microbiota were observed in T1D cases compared to healthy controls and to non-autoimmune diabetes cases such as Maturity Onset of Diabetes of the Young (MODY) (98).

Human studies conducted on diabetic patients have demonstrated the presence of dysbiosis that is associated with increased intestinal permeability and mucosal inflammation contributing to the development of islet autoimmunity (28, 99). In children with T1D, the microbiota is characterized by a low bacterial diversity, reduced microbiota stability, an increased amount of *Bacteroides, Clostridium* and *Veillonella* and a reduced number of Firmicutes (100, 101). Interestingly, *Bacteroides dorei* was found enriched in T1D-prone children before they developed autoimmunity (102). Moreover, at the onset of autoimmunity of ß-cells, the species *Bacteroides ovatus* was found 16 times more abundant in T1D cases than in the control group; several other *Bacteroides* species were enriched as well (103). In T1D patients a decreased abundance of *Prevotella, Bifidobacteria*, and *Lactobacillus* was reported; this led to reduced production of butyrate and lactate that have anti-inflammatory and immunomodulator functions and can improve the integrity of the mucosal barrier (104). The TEDDY study concluded that the microbiome in healthy controls resulted in a higher expression of genes related to fermentation and biosynthesis of short-chain fatty acids, that have a protective role against the development of autoimmunity. The healthy subjects had higher levels of *Lactobacillus rhamnosus* in their stool samples (105).

The mechanism of how the immune response is translocated from the intestine to the pancreas is still elusive. However, it is known that the mesenteric lymph nodes, which are part of the gut-associated lymphatic tissues (GALT), are linked to the pancreatic lymph nodes (106). Moreover, evidence shows that the intestinal immune response can be resembled in the pancreas by allowing T cells imprinted with homing receptors, that have adhesion molecules expressed in the pancreas, to infiltrate the pancreas and attack the islets cells (107).

## Early Nutrition As Modulator of Microbiota in Relation To T1D

Results from animal and human studies confirm that diet is one of the primary modulators of the gut microbial community also in T1D (108). A high carbohydrate (CHO) diet has been associated with a higher abundance of *Prevotella* (109) which was found reduced in T1D subjects (110, 111). Diets rich in fat increase the abundance of *Bacteroides* genus (109, 112), that was found enriched in children prone to develop T1D (102) as well at the onset of ß-cells autoimmunity in T1D cases (103).

The role of the gut microbiota significantly lies in the effect of the metabolites that they produce such as vitamins (113, 114), short-chain fatty acids (SCFA) (115–117), indole derivatives (118, 119) and organic acids (120). Consistently, high fiber diets yielding a high amount of butyrate and acetate, separately and combined, contributed to improved immune regulation and protection against T1D through different mechanisms. In diabetes-prone rats, although introducing butyrate during early life did not prevent T1D, it seemed to be associated with delayed T1D progression by reducing gut leaking compared to the control group (121). In humans, achieving a causal relationship among diet, SCFAs and T1D development is still challenging. Here below, we discuss the potential mechanism of early nutrition as modulator of microbiota in triggering T1D that remains not well elucidated.

### Breast-Feeding Effect on Microbiota of T1D Infants

In the past, the newborn babies were thought to have a sterile gut at birth; however, recent evidence suggests that the fetus is exposed to colonization of intrauterine bacteria (122). Several studies confirmed presence of bacteria in meconium samples in the 65–75% of infants with full-term delivery (123, 124). The variability of newborn microbiota is influenced by many factors such as the mode of delivery, vaginal delivery or cesarean section (122), and breastfeeding. The similarity between mother's milk bacteria and infant gut flora confirmed that microbiota can be transferred through breastfeeding. The diversity and the composition of infants' microbiota seem to be influenced by the amount and the duration of breastfeeding (125). The microbiota of infants exclusively breastfed for 6 months is enriched with *Bifidobacterium longum subsp. Infantis*, which is involved in child's immunity (126, 127). It belongs to lactic acid bacteria, *Bifidobacterium* and *Lactobacillus*, which can break down human milk oligosaccharides (128).

Former studies reported that the bacterial colonization was significantly different between breastmilk-fed and formula-fed babies (129–131). For instance, *Lactobacillus johnsonii/L.gasseri, L. paracasei*/*L. casei*, and *Bifidobacterium longum* are prevalent in exclusively breastfed infants whereas *Clostridium difficile, Granulicatella adiacens, Citrobacter spp*., *Enterobacter cloacae, Bilophila wadsworthia* are the most common bacterial species in the 4-months formula fed infants (132). Moreover, *E. coli* was found to be significantly lower in the gut microbiota of breastfed infants compared to formula-fed infants (133). Interestingly, Davis et al. described changes in the gut microbial composition upon shifting from breastfeeding to cow milk after 1 year and a half of life: *C. difficile* almost disappeared with a relative increase in *Bacteroides spp., Blautia spp., Parabacteroides spp., Coprococcus spp., Ruminococcus spp.*, and *Oscillospira spp*. and decrease of *Bifidobacterium spp., Lactobacillus spp., Escherichia spp., and Clostridium spp*. (134).

Breast feeding importance in modulating the gut microbiota in relation to T1D has been tackled in many studies with controversial results (8, 23). In breastfed infants, an abundance of *Bifidobacterium* was observed, which has been inversely correlated with T1D risk by a number of cross-sectional and longitudinal human studies (126, 128). *Lactobacillus* and *Bifidobacterium* species in breast milk have a protective role in preserving the gut integrity and stimulating the growth of Firmicutes bacteria phylum, that is found deficient in people with T1D (103, 135). The TEDDY study demonstrated a temporal development of the gut microbiome in T1D infants that can be divided in three phases: a developmental phase (months 3–14), a transitional phase (months 15–30), and a stable phase (months 31–46). Breastfeeding, either exclusive or partial, was associated with the microbiome temporal structure, with higher levels of *Bifidobacterium* species (*B. breve* and *B. bifidum*) at the development phase, and the cessation of breast milk resulted in faster maturation of the gut microbiome, marked by the phylum Firmicutes (136).

Furthermore, breast milk can be a source of insulin hormone, that enhances the gut microbiota diversity through the stimulation of the Gammaproteobacteria (137), which is crucial for infant gut microbiota maturation, especially during the first weeks of life (138). Leptin is another hormone contained in breast milk which is involved in beneficial bacterial metabolic pathways, such as stimulating microbial diversity and reducing bacterial proteases linked to intestinal permeability and inflammation (137). In addition, breast milk contains non-digestible oligosaccharides which promote the growth of beneficial bacteria, leading to a balanced microbiota (139). The beneficial effect of a breast milk rich in omega-3 fatty acids observed in the FINDIA study (36) can be explained with an involvement of the gut microbiota, as demonstrated in an animal model of type 2 diabetes. Animals fed with flaxseed oil, rich in omega-3, showed improved glycemic indexes, blood lipids, inflammatory cytokines and reduced levels of Firmicutes and *Blautia*, as well as an increase in the levels of Bacteroidetes and *Alistipes*. SCFA were also improved in the supplemented animals (140). No data are available on effect of omega-3 on gut microbiota of T1D patients.

Formula milk composition was also investigated in a multicenter double-blind clinical trial (from the FINDIA study), where 1,113 infants with HLA-conferred susceptibility to T1D were sorted into 3 groups and given respectively cow milk formula, hydrolyzed formula or bovine insulin-free formula during the first 6 months of life. The primary finding was that infant fed with insulin-free formula had a lower incidence of beta-cell autoimmunity by 3 years of age (141). In addition, an increase in *Bacteroides* and a decrease in *Bifidobacterium* amount were found in formula-fed infants who shifted to seroconversion (142).

### Complementary Feeding-Induced Microbial Changes and Immune Response

It is demonstrated that the diet composition (i.e., amount of fat, sugars, calories, vegetarian diet, etc.) affects the microbiota composition and the intestinal function (104). More importantly, nutrition in early life plays a critical role in the development of microbiota and the mucosal immune system starting from the breastfeeding habits to the introduction of solid food.

Early introduction of cow milk has been associated with increased intestinal permeability and gut inflammation (14). Children with T1D express higher levels of circulating antibodies against cow milk proteins (β-lactoglobulin, insulin, albumin); this could be the effect of a dysregulated immune response or an increased intestinal permeability (14). On another side, fermented milk products, i.e., yogurt, are frequently introduced at early stage as complementary food. Consumption of yogurt, kefir and other fermented dairy products are known for the health benefits mainly because of their probiotic effects on the gut flora (51). A systematic review summarized the beneficial findings of multiple studies performed in pediatric populations (0–24 months of age), discussed above, and it confirmed the hypothesis of the gut microbiota involvement to explain the improved diseases outcomes, particularly due to an increase in the *Bifidobacteria* genera (52). Animal studies have been performed to test the effect of fermented diary product on diabetes and cardiovascular biomarkers. A recent experimental obesity model showed improved insulin sensitivity and cardiometabolic risk factors in obese mice fed with Streptococcus-fermented milk or Greek-style yogurt, modulating the gut microbiota composition and the intestinal immune response (143). A second animal study on diabetic rats fed with yogurt fermented with *Lactobacillus casei* showed improved blood glucose and insulin level and reduced expression of genes involved in the liver gluconeogenesis, together with a shift in the microbiota composition and SCFA abundance (144). A human study testing the effect of fermented dairy on the Trimethylamine-*N*-oxide (TMAO) metabolites of the gut microbiota associated to the cardiovascular risk, demonstrated that healthy young men consuming yogurt or acidified milk for 2 weeks showed decreased levels of TMAO and different microbiome composition (145). To the best of our knowledge, no studies have been performed on the effect of fermented dairy in T1D subjects or animal models.

Complementary feeding is associated with a modification in the gut microbiota composition, influenced by proteins and fibers from solid foods. Non-digestible carbohydrates contained in solid foods provide substrates for the development of specific microbes (146). During the complementary feeding period, there is a reduction in *Bifidobacteriaceae, Lactobacillaceae, Enterobacteriaceae*, and *Enterococcaceae* and an increase of *Clostridium coccoides, Lachnospiraceae, Ruminococcaceae, Bacteroidaceae*, and *Sutterellaceae* (147, 148). However, *Bifidobacteria*, together with *Bacteroides*, remain among the prevalent species found in children's microbiota even after the introduction of complementary feeding (146). A recent study showed that the early introduction (<3 months) of complementary food is associated with an altered microbial composition with a higher diversity and an accelerated maturation pattern (149). Increased fecal excretion of butyrate and other SCFAs is associated with both local and systemic effects. It is a sign of a reduced microbial diversity and an increased gut barrier permeability, and it is associated with systemic inflammation, hyperglycemia, dyslipidemia and an increased risk of developing obesity and hypertension (150). Moreover, the lower level of *Bifidobacterium*, which plays a key role in digesting oligosaccharides and producing vitamins and SCFAs, may also contribute to inflammation. Instead, the greater abundance of *Akkermansia Muciniphila* promotes an early growth of adult associated bacteria, impairing the growth of taxa typical of the developmental phase. Finally, the early introduction of food results in an increased concentration of fecal butyrate (149).

### Gluten and Cereals Modulate the Gut Microbiota in T1D

A diet rich in fat and sugar like the “western diet” leads to an increase in *Clostridium innocuum, Eubacterium dolichum, Catenibacterium mitsuokai*, and *Enterococcus spp*. and a reduction in *Bacteroides spp*. (151). A carbohydrate-reduced diet or a low-caloric diet can revert the trend as observed in mice (152). The western diet also reduces the growth of other species such as *Clostridium coccoides, Lactobacillus spp*., and *Bifidobacteria spp*.; however, a diet rich in complex carbohydrates enhances the reduction of *Mycobacterium avium* subspecies and *Enterobacteriaceae* and the increase in *Bifidobacteria spp*. (153). Protein-rich diets can stimulate the activity of bacterial enzymes (β-glucuronidase, azoreductase, and nitroreductase); this leads to the production of toxic metabolites that promote inflammation (154). On the contrary, the vegetarian diet, which is rich in fibers, stimulates the short chain fatty acids' production, causing a decrease in the intestinal pH and preventing the overgrowth of pathogenic bacteria (104, 155, 156).

The link between dietary gluten and T1D is gaining the interest of researchers. The concomitant presence of T1D and celiac diseases is not rare (8% of coexistence) and it is challenging the clinicians with complex immunological and clinical management. At the date, the insulin and a gluten-free diet (GFD) are still the only recommended treatments for T1D and the celiac disease, respectively. However, these treatments pose certain challenges to both the clinicians and the patients, as GFD has a high glycemic index that affects the glycemic control. Moreover, intermittent gluten intake by these patients due to non-compliance with GFD stimulates the autoreactive immune cells which in turn result in an augmented immune response (157). A randomized controlled trial adopting GFD for 1 year on T1D and subclinical celiac disease patients, showed a decrease number of hypoglycemic episodes and a better glycemic control (158). Conversely, an observational case-control study on T1D and GFD-treated celiac patients compared with T1D alone showed that a long-term GFD does not affect the glycemic control, but it has a different impact on diabetes complications (159). The evidence is still unclear regarding the dietary approach for T1D and celiac patients, yet selected gluten free food high in fibers can better control the glycemia (160). Further studies and clinical trials are needed to test different types of GFD and gluten free foods. Moreover, other factors need to be included in the discussion such as the gut microbiota.

Concerning the role of gluten on the gut microbiota, no specific study has been conducted. However, in the latest years many studies have been published on the role of microbiome in the development of celiac disease (CD), resulting in heterogeneous data. Children affected by celiac disease have microbial dysbiosis, and this is thought to contribute to the pathogenesis of CD. The function of Th17 cells, that play a role in the inflammatory response against gliadin peptides, is influenced by the microbiome (161). Children with CD has a microbiome characterized by a higher amount of Proteobacteria, Bacteroides, Actinobacteria, *Neisseria spp.*, and *Haemophilus spp*. and a lower abundance of *Lactobacillus* and *Bifidobacterium* (162). The gluten-free diet can restore the microbiome composition only partially and the reasons are obscure; a possible hypothesis could be the influence of genetic background on the microbial composition (163). Moreover, a gluten free diet in healthy subjects has been associated with a decrease of “healthy bacteria” (*Bifidobacterium, Lactobacillus, Roseburia*) and an increase of potentially unhealthy bacteria (*Enterobacteriaceae, Clostridiaceae*) (164). Therefore, based on the available data, it is not possible to draw any conclusion about the role of gluten on the T1D associated to gut microbiota changes.

The use of *Bifidobacterium* strains as probiotics showed a reduction in the gut inflammation and the production of proinflammatory cytokines which consequently improve the gut barrier's function. It was also demonstrated that probiotics can reduce the toxicity of gliadin by degrading the proinflammatory gluten peptides and reducing their immunogenicity (165).

### Micronutrients Intake and the Effect on the Gut Microbiota in T1D

Micronutrients are able to interfere with the function of the microbiota (166). No studies investigated the effect of micronutrients deficiency on the gut microbiome of T1D patients. However, many evidences are available of the role of micronutrients in affecting microbiota in early life and in similar disease setting.

Vitamin D in particular plays a pivotal role in intestinal integrity, intestinal immunity and microbiome modulation in the autoimmune diseases, assuming a common mechanism of action affecting the immune response and the gut permeability (167). Microbial composition has been found affected by the season and directly correlated with vitamin D levels in Inflammatory Bowel Disease (IBD) patients (168). No data were found directly measuring the effect of vitamin D on gut microbiota of T1D patients, but we can speculate on similar mechanisms because of the frequent comorbidity of T1D and IBD (169).

Vitamin A and its receptor are involved in the response to pathogens modulating the response of IL-18 and IFN-γ (170) and the gut microbiota composition (166), but no studies have been performed to investigate the effect of vitamin A on the infant microbiota of T1D patients. One study tested the effect of vitamin A supplementation within 48 h of life on healthy group of newborns and measured the effect on the gut microbiota at 6–15 weeks of life and later at 2 years of age. The authors found a difference between gender where the boys were more responsive to the treatment with increased abundance of the *Bifidobacterium* compared to the girls in early infancy, and instead girls at 2 years of life showed an increased level of *Akkermansia* (171), concluding on a potential contribution of vitamin A in the development of a heathy gut microflora.

SunGold kiwifruit, containing high levels of vitamin C, was tested on prediabetic people and the daily consumption over 12 weeks, even if it improved metabolic and anthropometric parameters, slightly impacted on the gut microbiota (172). An animal model was used to investigate the effect of antioxidant blends supplementation, including vitamin C, in piglets at the weaning age. The supplementation was able to restore the unbalanced antioxidant capacity, the intestinal levels of *Lactobacillus* and *Bifidobacterium* and to reduce free radicals and levels of *Escherichia coli* caused by the early weaning stress (173).

Vitamin E and iron supplementation in a group of iron-deficient infants and toddlers showed an increase of *Roseburia* compared to subjects supplemented with iron only (174). The effect of iron supplementation on the gut microbiota is not clear. Many studies have been performed with discrepant results due to different iron doses, chemical formula and administration routes (166).

Data on minerals and gut microbiota are also limited. Animal studies showed that the selenium supplementation increases the microbial diversity, and that the many species are selenium-dependent competing with the host on the selenium availability (175) and contributing to the metabolism of the seleno-proteins (176). Zinc and copper supplementation in pigs at the weaning age showed also decrease levels of *Streptococcus, Enterobacter* and *Escherichia*, and increased levels of *Lachnospira* and *Roseburia* (177). No studies are available on the effect of minerals on the gut microbiota of children affected by T1D, however there are clear evidences of micronutrients deficiencies in T1D adult population (178) and of the beneficial effect of antioxidant vitamins C and E on endothelial function in T1D cohort (179). Further studies are needed to investigate the effect of micronutrients deficiencies and potential supplementation during transition to solid foods to reduce the risk of T1D and its complications.

## Probiotics/Prebiotics/Postbiotics Protective Effect Against Autoimmunity

Given the influence of altered microbiota in the development of the disease, an increasing number of studies evaluated the chance to modulate microbiota through dietary interventions or probiotic supplementation, in an attempt to induce a more tolerogenic environment and reduce the risk of islet autoimmunity and diabetes (16, 100).

Results from experimental studies and clinical trials support that the modulation of gut microbiota by probiotics administration may be protective toward T1D through several mechanisms: probiotics can increase the expression of junction and adhesion proteins, promote the barrier's function, reduce the oxidative stress and modulate the inflammatory response which lead to increased number of T-regulatory cells and anti-inflammatory cytokines (99). NOD mice supplemented with probiotics have a lower degree of insulitis (93). Another study, conducted in the USA on diabetic prone rats, showed that post-weaning administration of *Lactobacillus johnsonii* N6.2 reduces or delays the onset of T1D, by influencing the microbiota, the intestinal proteins and the oxidative stress. Rats that received probiotics had lower IFNγ, INOs, and occluding, a higher amount of claudin, COX 2, and an increased expression of Globet cells. This leads to a reduction in the inflammation and the oxidative stress and an improvement in the intestinal barrier integrity. The same effect was not observed with the supplementation of *Lactobacillus reuteri* (95). An Italian study showed that supplementation with *Lactobacillaceae*-enriched probiotic VSL#3, given alone or along with retinoic acid (RA), decreases the risk of developing diabetes in non-obese diabetic (NOD) mice (93). The microbiome profile of NOD mice supplemented with VLS#3 is characterized by a reduced amount of *Bacteroides* and increased quantity of *Lactobacillaceae, Clostridia* (promoting FoxP3+ Treg cell differentiation in the intestinal mucosa) and *Rikenellaceae*. In addition, probiotic VLS#3 reduces the expression of proinflammatory cytokine IL-1β and increases the production of pro-tolerogenic and immunomodulatory factors, such as indoleamine 2,3-dioxygenase (IDO) and IL- 33. Moreover, VLS#3 stimulates the differentiation of tolerogenic dendritic cells (CD103+) and limits the expansion and the differentiation of inflammatory T-cells (Th1 and Th17) in the intestinal tract, the spleen and the pancreatic lymph nodes. The combination of VLS#3 with RA induces proliferation of T-regulatory cells in the intestinal mucosa. VLS3# also increases the expression of zonulin-1 in the intestinal mucosa, promoting the integrity of the intestinal barrier (93). The TEDDY study showed that early probiotic administration (<27 days of life) is associated with a reduced risk of islet autoimmunity compared to a later or any supplementation (180). By contrast, a Finnish study could not find any correlation between probiotic supplementation and the development of islet autoimmunity by 5 years, not even with T1D progression by 13 years (181). However, results from clinical trials depend on specific bacterial strains used as probiotic and cannot be applied to other species.

Prebiotics are non-digestible carbohydrates that can have a role in modulating the immune system and the gut microbiome, contributing to the development of autoimmune diseases such as T1D (182). Studies conducted on animals and humans showed a positive effect of prebiotics' supplementation on the microbiome and the immune system that can reduce intestinal permeability and inflammation, reducing the risk to develop T1D (183). A study conducted on NOD mice showed that long-term oral supplementation of low-dose β-glucan derived from yeast modifies the microbiota composition by reducing the amount of Firmicutes and increasing the abundance of Bacteroidetes phylum, Verrucomicrobia phylum and the polysaccharide-fermenting bacteria. The β-glucan supplementation increases also some metabolic pathways, such as the carbohydrates metabolism and the glycan biosynthesis and metabolism. The use of this prebiotic has also an immunomodulatory effect, with an increased number of T-regulatory cells in the intestinal wall, favoring the development of immunotolerance, which reduces the degree of insulitis and the subsequent risk of developing T1D (184). A pilot study on T1D pediatric patients using administration of oligofructose-enriched inulin are on-going, expecting to improve the glycemic control modulating the gut microbiota and the intestinal permeability (185). The immunomodulatory effect of prebiotics could lead to consider the possibility of introducing their supplementation in infants in order to reduce the risk of developing T1D.

A different strategy to prevent the development of autoimmunity and T1D can be the supplementation of microbial products (also known as post-biotics), such as SCFA, in order to modulate the microbiota and the immune system. The SCFAs (acetate, propionate and butyrate) have indeed an anti-inflammatory and immunomodulatory function. They promote the T-regulatory cells function, modulate cytokines' production, increase the expression of anti-microbial peptides, reduce oxidative stress and regulate the epithelial barrier function by increasing the expression of tight junction and the production of mucin (28). A study conducted on NOD mice confirmed the protective role of a combined acetate- and butyrate-yielding diet against diabetogenic pathways by decreasing the autoimmune response and boosting the function of regulatory T cells (186).

The probiotic, prebiotic and post-biotic therapeutic approach looks extremely interesting, but it still requires more clinical trials to confirm the efficacy and specificity of the treatment in T1D patients of different age groups, with different environment, diets and genetic backgrounds.

## Conclusions

**Figure 1:**
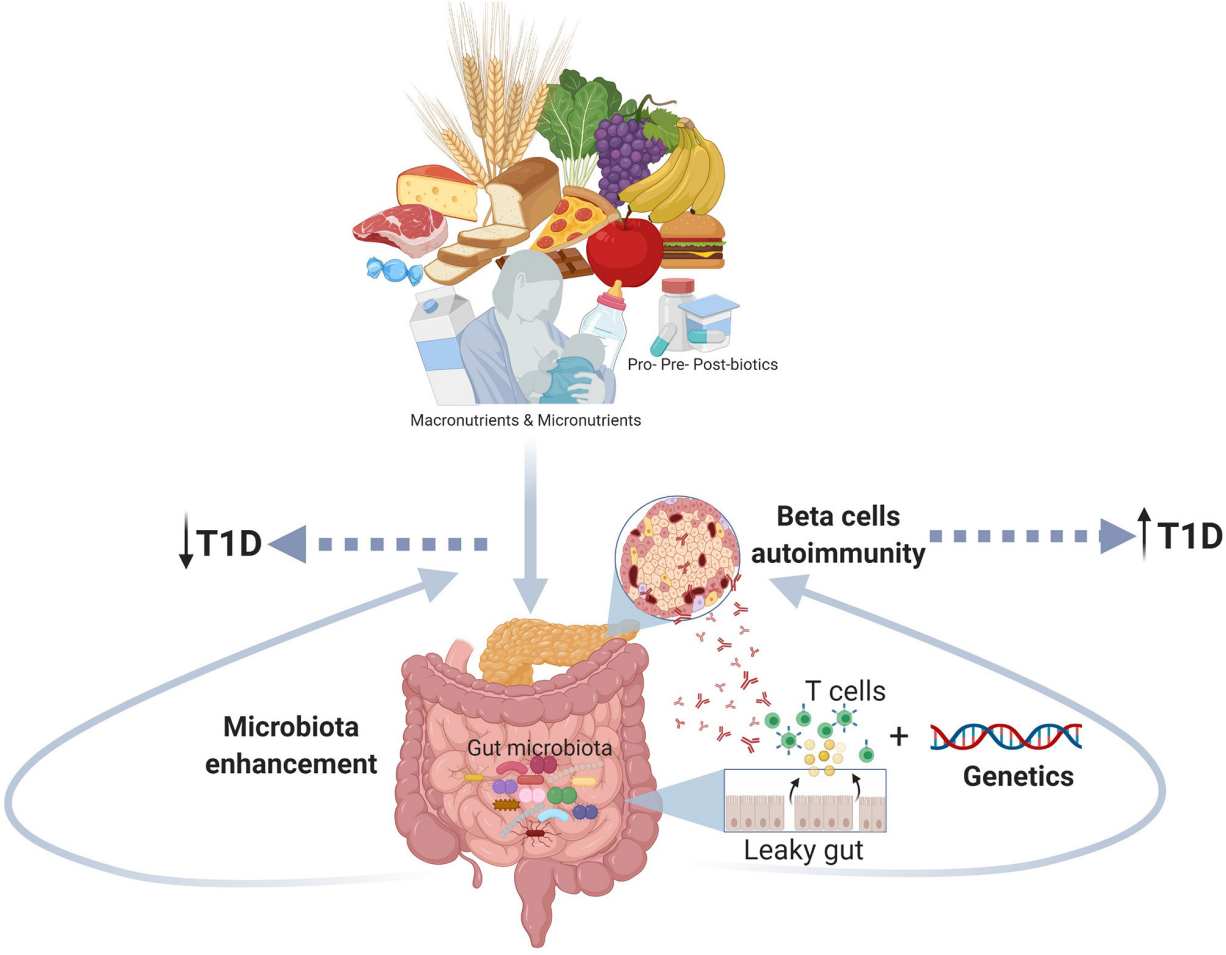
The figure shows the role of the early nutrition in improving a healthy gut microbiota and counteracting gut dysbiosis and autoimmune response as potential mechanism involved the pathogenesis of T1D (Created using Biorender.com).

The T1D is a complex disorder with an unclear etiopathogenesis and an increased incidence in the last decades that presumes the contribution of multiple factors: genetics, age, environment, and diet. In this review, we focused our attention on the role of early diet as a modular of the gut microbiota and, indirectly, of the immune system (Table 1). We hypothesize a mechanism where long-term breastfeeding and avoiding early (<4 months) introduction of solid foods, associated with a diet rich in micronutrients, contribute to developing healthy gut microbiota that boosts the maturation of the immune system and hence reduce the risk of T1D (Figure 1). The usage of probiotics, prebiotics and post-biotics in the prevention of T1D also gained great interest, yet it still requires further studies that will define the precise microbial profile of different types of patients (age, ethnicity, environment, diet, etc.) and test the specific treatment of each type of T1D microbial profile.

**Table 1:** Summary of the dietary factors affecting gut microbiota and their direct or indirect role in T1D.

**Dietary factors**	**Type of study**	**Findings**	**References**
Breast milk	Human studies (T1D infants in the first 3 years of life)	Increased abundance of *Bifidobacterium* inversely correlated with T1D risk	(126)
	Human study (T1D infants 3–46 months of age)	Increased abundance of *Bifidobacterium*	(136)
	Human studies (infants at 2 weeks of life)	Insulin and leptin hormones in the breast milk increased microbial diversity and abundance of Gammaproteobacteria	(137)
Formula milk	Human clinical trial (infants 0–6 months of age at risk of T1D)	Insulin-free Formula milk Decreased AI	(141)
	Human clinical trials (infants 0-8 months of age at risk of T1D)	Positive association of AI with *Bacteroides* and negative association with *Roseburia*	(142)
Cow milk	Human studies (T1D children)	Early introduction of cow milk association with increased gut permeability	(14)
Fermented milk products	Human study (infants 0–24 months of age)	Increase in the *Bifidobacterium*	(52)
	Obese animal model	Improved insulin sensitivity, increased abundance of *Streptococcus*, improved immune response	(143)
	Diabetic animal model	Improved glycemic indexes and shift in microbiota and SCFA composition	(144)
Complementary food	Human study (infants 0–12 months of age)	Early introduction (<3 months of life) was associated with accelerated maturation and higher diversity of the gut microbiota and increased levels of SCFA	(149)
Gluten and Cereals	Randomized clinical trial on CD–T1D adult patients	GFD improved glycemic control	(158)
	Observation case-control study on CD–T1D adult patients	GFD affects diabetes complications	(159)
	Observational case-control study on CD pediatric patients (5 years old)	GFD can partially restore microbiota	(163)
	Clinical trial on healthy adults	GFD decreases *Bifidobacterium* and *Lactobacillus*, levels of SCFA and affect the immune response	(164)
omega-3 PUFA	T2M animal model	Improved glycemic indexes, inflammatory status, increased levels of Bacteroides and SCFA, reduced levels of Firmicutes	(140)
Vitamin D	Observational study on IBD adult patients	Seasons affect levels of Vitamin D and microbiome composition	(168)
Vitamin A	Interventional study on healthy newborns (effects measured at 6–15 weeks and 2 years of age)	Increased levels of *Bifidobacterium* at 6–15 weeks old boys; increased levels of *Akkermasia* in 2 years old girls	(171)
Vitamin C	Interventional study on prediabetic adult population	Improved metabolic parameters and slightly affected microbiota	(172)
	Antioxidants supplementation on animal model at weaning age	Increased levels of *Bifidobacterium* and *Lactobacillus*, reduced level of *E. Coli*, improved antioxidant capacity	(173)
Vitamins C and E	Interventional study on T1D adolescents (mean age 12 years old)	Improved endothelial function	(179)
Vitamin E and iron	Interventional study on iron-deficient infants and toddlers	Increased levels of *Roseburia*	(174)
Zinc and Copper	Supplementation on animal model at weaning age	Decreased levels od *Streptococcus, Enterobacter, Escherichia*, increased levels of *Lachnospira* and *Roseburia*	(177)
Probiotics	T1D animal model	*Lactobacillus johnsonii* N6.2 induced a delay in T1D onset, improved intestinal epithelial function, reduced the inflammation, modulated microbiota	(95)
	T1D animal model	*Lactobacillae-*enriched probiotic VSL#3 decreased the risk of T1D, reduced amount of *Bacteroides*, increased amounts of *Lactobacillae, Clostridia* and *Rikenellaceae*, improved immune response and intestinal epithelial function	(93)
	Interventional studies on T1D infants (<27 days of life)	Probiotic administration reduced the risk of islet AI	(180)
	Interventional studies on T1D infants (0–6 months of life)	No correlation of probiotic mixture administration with the risk of islet AI and diabetes progression	(181)
Prebiotics	T1D animal model	β-glucan supplementaion reduced levels of Firmicutes increased Bacteroidetes, Verrucomicrobia and polysaccharide-fermenting bacteria, improved carbohydrates metabolism, improvef immune function, reduced insulitis degree	(184)
	Interventional studies on T1D children (8–17 years old)	Study on going. Expected to improve intestinal permeability and glycemic control	(185)
Post-biotics	T1D animal model	Protective role of SCFA, reduced AI, improved immune function	(186)

*AI, auto immunity; SCFA, short chain fatty acids; CD, celiac disease; GFD, Gluten-free diet*.

The key messages from this paper are that promoting long-term breast-feeding during the first 6 months of life, avoiding early complementary foods and gluten introduction (before 4 months of age), and avoiding cow milk introduction before 12 months of life, may reduce the risk of developing T1D. The gut microbiota is affected by early nutrition, and the gut microbiota dysbiosis in infancy may contribute to the onset of T1D. Therefore, there is a need to plan randomized clinical trials testing the role of probiotics, prebiotics and post-biotics in T1D prevention.

## Author Contributions

AT and EV planned the manuscript and discussed the content. CM and AP wrote the introduction and about the clinical aspect of T1DM. SV and MA wrote the sections on early nutrition and probiotics. SA and FE wrote the microbiome sections and formatted the references. SA created the figure and formatted the final version of the manuscript. AT and EV reviewed and finalized the manuscript. GZ supervised and reviewed the manuscript. All Authors approved the submitted version of the manuscript.

## Conflict of Interest

The authors declare that the research was conducted in the absence of any commercial or financial relationships that could be construed as a potential conflict of interest.
